# Efficient derivation of hiPSC-derived photoreceptor precursor cells and their neuroprotective effects in retinal degeneration

**DOI:** 10.1016/j.isci.2025.114196

**Published:** 2025-11-24

**Authors:** Yuxin Du, Jingjing Cao, Lumeng Niu, Gao Tan, Jingmin Zhang, Xiaoqian Yi, Yu Li, Jun Wei, Yin Shen

**Affiliations:** 1 Eye Center, Renmin Hospital of Wuhan University, Wuhan, Hubei 430060, P.R. China; 2 Zhongmou Therapeutics Co., Ltd., Wuhan, Hubei 430060, P.R. China; 3 iRegene Therapeutics Co., Ltd, Chengdu, Sichuan 610200, P.R. China; 4 Frontier Science Center for Immunology and Metabolism, Medical Research Institute, Wuhan University, Wuhan, Hubei 430071, P.R. China

**Keywords:** molecular biology, neuroscience, cell biology

## Abstract

Retinal degenerative diseases such as retinitis pigmentosa (RP) lack effective therapies capable of restoring vision. Photoreceptor precursor cell (PPC) transplantation is a promising regenerative strategy. This study utilized the PPCs efficiently derived from human-induced pluripotent stem cell (hiPSC). The employment of AM580, a chemical compound of oxidatively stable retinoid analogs, facilitated the differentiation of hiPSCs into CRX^+^/LHX4^+^ PPCs with 99.9% purity. Furthermore, the retinal protective effect of PPCs was confirmed by the result of a bi-dimensional increase in intensity and space in the N-methyl-N-nitrosourea-induced RP model. Besides the retinal protective effects, donor PPCs were observed to express rod- and cone-specific markers and to develop CtBP2^+^ presynaptic specializations that were located in close proximity to host bipolar cells. These observations are consistent with the ability of hiPSC-derived PPCs to engage in structural repair processes, supporting further investigation into their potential for treating retinal degeneration.

## Highlights

• AM580-based protocol rapidly generates hiPSC-derived PPCs with >99.9% purity
• Transplanted PPCs mature, express opsins and form presynaptic specializations
• PPC transplantation provides neuroprotection, preserving retinal structure in an RP model

## Graphical abstract

**Figure:**
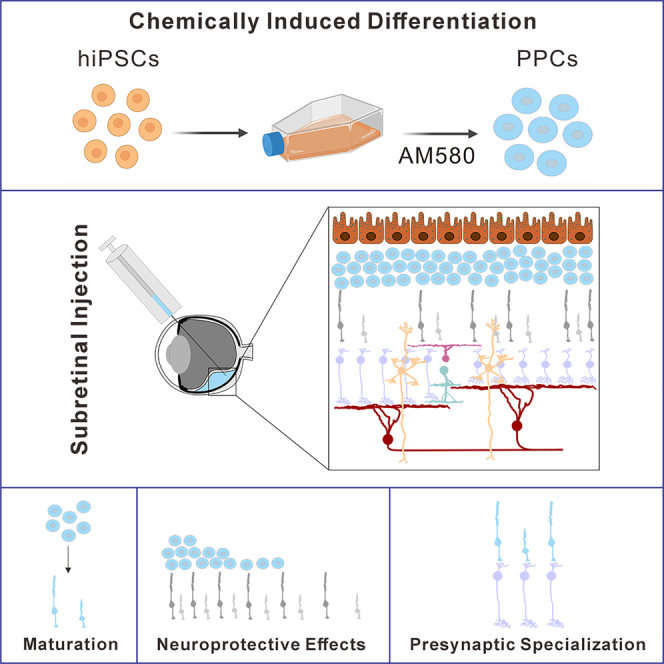

## Introduction

Retinal degenerative diseases, a group of clinically irreversible blinding conditions, constitute major therapeutic challenges in modern ophthalmology.^1^ Approximately 285 million people worldwide are affected by these disorders.^2^ Retinal degenerative diseases include retinitis pigmentosa (RP), age-related macular degeneration (AMD) are unified by a core pathophysiological mechanism, which is progressive photoreceptor dysfunction culminating in irreversible cellular loss. In advanced stages, photoreceptor depletion limits the therapeutic outcomes of traditional gene therapies, which require residual photoreceptors as transduction targets.^3^ There has been a concerted global focus on alternative strategies for achieving functional vision restoration. Such strategies include, but not limited to optogenetics, photoswitches, retinal prostheses, and photoreceptor cell transplantation.^4^^,^^5^^,^^6^

Photoreceptor transplantation is a regenerative approach that aims to restore retinal circuitry by replenishing lost photoreceptors, which are specialized neurons converting light into neural signals. The substantial evidence has validated its therapeutic safety and efficacy for retinal degenerative diseases.^7^^,^^8^^,^^9^ Nevertheless, critical challenges persist regarding donor cell sources, particularly ethical considerations, manufacturing scalability, safety profiles, and long-term functional efficacy. Optimization of donor cell types and manufacturing platforms is a prerequisite for achieving robust cell survival, host-graft integration, and functional recovery.^10^ The advent of innovative cell culture technologies, including two-dimensional (2D) and three-dimensional (3D) systems, are enabling photoreceptor cell production toward clinical application.

Despite the technical simplicity and cost-effectiveness of 2D culture, it was reported that 2D culturing was accompanied by low differentiation efficiency or long-term differentiation.^11^^,^^12^ Recent breakthroughs in 2D differentiation protocols have enhanced retinal lineage specification from human iPSCs (hiPSCs). The ectoderm differentiation strategy that is based on small molecules has its origins in dual SMAD inhibition.^13^ Mechanistically, SB431542 (TGF-β receptor/ALK5 inhibitor) and LDN193189 (BMP receptor/ALK2/3 inhibitor) synergistically promote neuroectodermal specification via embryonic signaling pathway modulation.^13^^,^^14^ This paradigm enables reproducible generation of retinal pigment epithelium (RPE) and photoreceptor precursor cells (PPCs) without requiring transwell cultures or organoid systems.^15^ Notably, the standard dual SMAD inhibition with small moleculer cocktails IWR1, SB431542, and LDN193189, combining with growth factor IGF1, has been reported to generate OTX2^+^ cell within 12 weeks.^16^^,^^17^ Remarkably, xenotransplantation of hESC-derived retinal cells into murine subretinal space (SRS) induces mature photoreceptor marker expression accompanied by functional integration within 3 months post-transplantation.^18^ Alternative protocols employing sequential small molecule induction (e.g., fasudil, nicotinamide, noggin, DKK-1, IGF-1, FGF2, activin A, SU5402, and CHIR99021) demonstrate co-expression of RPE (MITF^+^) and photoreceptor (CHX10^+^) markers during 14-day neural induction.^19^ Furthermore, a xeno-free system utilizing human recombinant laminin LN523 accelerates PPC differentiation to 32 days through three-phase induction (neural induction: SB431542+CKI-7; photoreceptor specification: BDNF/CNTF/RA/DAPT).^20^

Conventional dual SMAD inhibition strategies incorporating serum components and retinoic acid (RA) suffer from prolonged differentiation (>70 days) and biohazard risks (animal-derived contaminants, allergenicity).^13^ Moreover, RA’s photolability and oxidative instability necessitate serum co-supplementation, complicating clinical translation.^21^ Herein, we engineered a serum-free induction system substituting RA with oxidatively stable retinoid analogs AM580. This platform eliminates zoonotic risks while achieving high-purity photoreceptor production from pluripotent stem cells. Subretinal transplantation in rodent RP models elicited donor cell survival, structural maturation and the formation of synaptic-like structures in contact with host neurons.

## Results

### Generation of PPCs from hiPSCs via small molecule-driven differentiation

**Figure 1:**
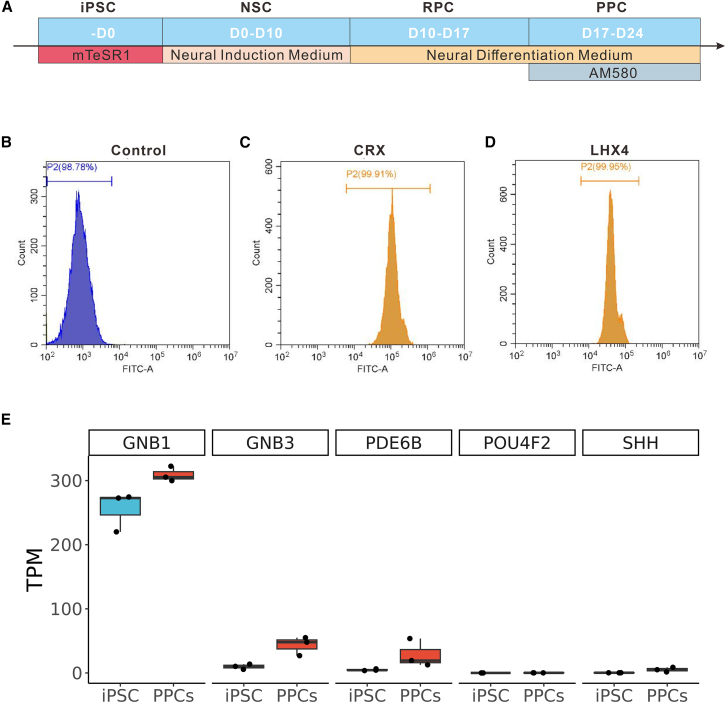
Differentiation and characterization of hiPSCs into PPCs (A) Schematic representation of the three-stage chemically defined differentiation protocol for generating PPCs from hiPSCs. Stage 1 (day 0 to day 10): HiPSCs induction using Neural Induction Medium to generate NSCs. Stage 2 (day 10 to day 17): RPC specification via Neural Differentiation Medium. Stage 3 (day 17 to day 24): terminal differentiation into PPCs using Neural Differentiation Medium supplemented with AM580. (B) Flow cytometry analysis of PPCs derived from hiPSCs. Unstained cells served as the negative control. (C) CRX expression in PPCs, as determined by flow cytometry. (D) LHX4 expression in PPCs, as determined by flow cytometry. (E) Expression levels (transcripts per million, TPM) of photoreceptor markers (GNB1, PDE6B, and GNB3) and RGC markers (POU4F2 and SHH) in undifferentiated iPSCs versus PPC-derived populations harvested at day 54 of differentiation (*n* = 3). Photoreceptor markers show robust induction in PPCs, while RGC markers remain undetectable. Data confirm progression toward advanced photoreceptor maturation, aligning with late retinogenesis trajectories. Data are presented as mean ± SEM.

HiPSCs were grown in Matrigel-coated 6-well plates until 80%–90% confluent, followed by Accutase-mediated dissociation to initiate differentiation (Figure 1A). Neural induction was performed using NouvNeu Neural Induction Medium for hiPSCs specification into neural stem cells (NSCs) from day 0 to day 10. Retinal progenitor cell (RPC) specification was then achieved using NouvNeu Neural Differentiation Medium from day 10 to day 17. Subsequent photoreceptor commitment was performed using NouvNeu Neural Differentiation Medium supplemented with 0.5 μM AM580 (a retinoic acid receptor α agonist) from day 17 to day 24 (Figure 1A). Differentiated PPCs were cryopreserved in STEM-CELLBANKER medium for long-term biobanking and downstream applications.

Flow cytometry at the terminal differentiation timepoint (day 24) demonstrated robust lineage commitment, with 99.91% CRX^+^ and 99.95% LHX4^+^ populations (Figures 1B–1D). CRX^+^ is correlated to photoreceptor commitment,^22^ while LHX4 is reported to be required for retinal rod bipolar cell differentiation.^23^ CRX^+^ and LHX4^+^ confirmed photoreceptor identity of PPCs.

The cellular specimen was harvested at day 24 of differentiation for single-cell RNA sequencing (scRNA-Seq) analysis. To elucidate the developmental progression of PPCs (presumptive photoreceptors), comparative analysis was performed against the established human fetal retinal development scRNA-seq reference atlas.^24^ ScRNA-Seq analytical outcomes demonstrated that which showed that PPCs at day 24 express early RPC markers (e.g., SFRP2) and lack strong expression of late-stage photoreceptor markers such as GNB3.^24^^,^^25^ PPCs occupied an earlier developmental niche relative to fetal retinal reference specimens, exhibiting incomplete differentiation toward mature retinal cell lineages as defined in the comparative atlas. Consequently, these cells displayed insufficient transcriptional congruence with established fetal subpopulations, precluding definitive mapping to any characterized cell type within the reference developmental framework (Figure S1).

To resolve this ambiguity in developmental trajectory and definitively assess lineage commitment, we performed Bulk RNA sequencing (Bulk RNA-seq) on PPC-derived populations at day 54. Transcriptomic profiling revealed robust induction of terminal photoreceptor markers (including GNB1, PDE6B, and GNB3),^25^ concurrent with the absence of canonical retinal ganglion cell markers (POU4F2 and SHH; Figure 1E). This later-stage expression signature demonstrated high concordance with transcriptional programs characteristic of late human retinogenesis.^24^^,^^25^ Collectively, the bulk transcriptome data confirm that early PPCs (day 24) progress along a photoreceptor lineage trajectory, culminating in a molecular profile consistent with advanced photoreceptor differentiation. This complementary approach resolves limitations in cell identity assignment from early single-cell data alone and validates the PPCs’ developmental potential.

### Temporal dynamics of stage-specific markers during hiPSCs differentiation into PPCs

**Figure 2:**
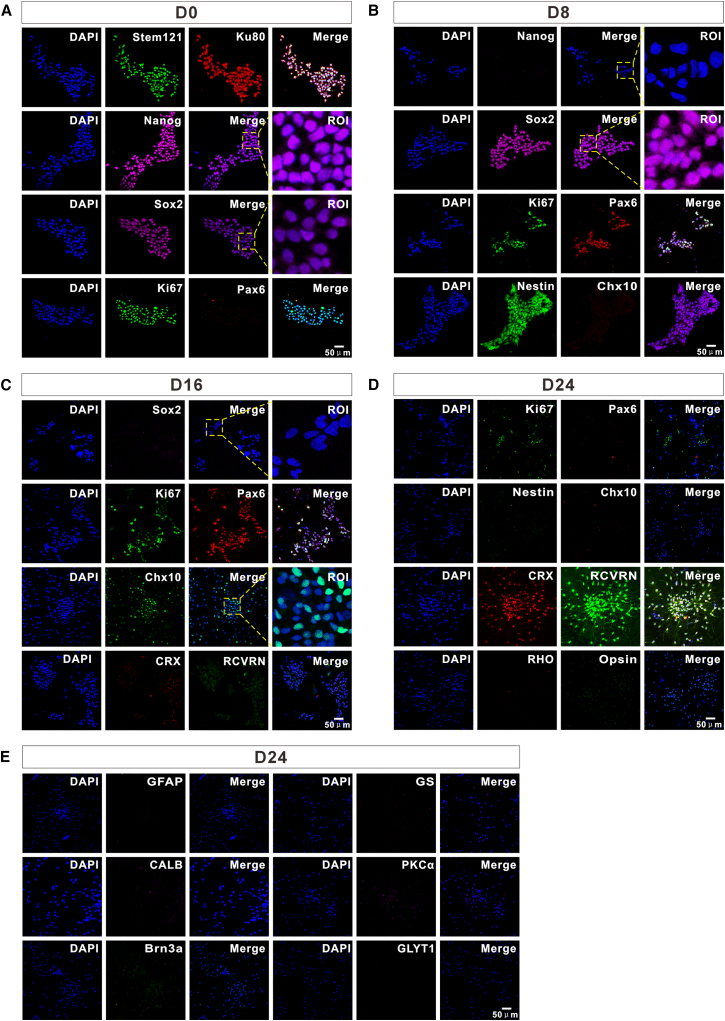
Temporal lineage marker analysis of hiPSCs differentiation into PPCs (A) Immunofluorescence staining of hiPSCs on coverslips at day 0. Cells were positive for human cytoplasmic marker Stem121 and nuclear marker Ku80, confirming their human origin. Robust nuclear expression of the pluripotency core factor Nanog and Ki67 positivity indicated active proliferation. Pax6, an NSC marker, was negative, while Sox2 positivity suggested potential transition toward the neural ectodermal lineage. (B) Immunofluorescence staining of cells at day 8 of differentiation. Nestin, a neural precursor intermediate filament protein, was positive, while Chx10, an RPC marker, remained negative. Nanog expression was significantly downregulated, and Ki67-positive cell proportions decreased, indicating suppression of pluripotency and initiation of lineage commitment. Sox2 nuclear signals were observed. (C) Immunofluorescence staining of cells at day 16 of differentiation. Nuclear expression of Pax6 (a core RPC regulator) and Chx10 (a neural retina lineage-specific marker) confirmed RPC specification. Ki67-positive cell proportions further decreased, though residual Ki67 signals in Pax6-positive cells indicated transient proliferative subpopulations. CRX and RCVRN, markers of photoreceptor fate, were undetectable, and Sox2 expression was completely silenced. (D) Immunofluorescence staining of cells at day 24 of differentiation. PPCs exhibited robust nuclear CRX expression and cytoplasmic RCVRN positivity, marking terminal differentiation. However, rod-specific RHO and cone-specific L/M Opsin were undetectable, indicating delayed photopigment expression. Chx10 and Nestin were negative, confirming exit from progenitor states. Minimal Ki67 expression suggested cells were in the final stages of differentiation. (E) Immunofluorescence staining at day 24 to exclude non-photoreceptor lineages. Cells were negative for Müller cell markers (GFAP and GS), HC marker (Calbindin), BC marker (PKCα), RGC marker (Brn3a), and AC marker (GlyT1). Regions of interest (ROIs). Scale bars represent 50 μm.

Through multiple time point immunofluorescence analysis of cells on coverslips, we systematically delineated the time course of hiPSCs differentiation into NSCs, RPCs, and PPCs under chemical induction. We note that retinal development in mammals follows a conserved spatiotemporal sequence: pluripotent stem cells transition to neural ectoderm (Sox2^+^/Nestin^+^), then commit to retinal progenitor fate (Pax6^+^/Chx10^+^), and finally differentiate into photoreceptor precursors (CRX^+^/RCVRN^+^) before maturing into rods/cones (RHO^+^/L/M Opsin^+^).^26^ Our protocol recapitulates key milestones of this *in vivo* process within a defined 24-d timeline. Cells harvested at differentiation days 0, 8, 16, and 24 were analyzed for marker expression. At the initial stage (day 0), hiPSCs exhibited robust nuclear expression of the core pluripotency factor Nanog and widespread Ki67 positivity, indicating active proliferation, while Sox2 positivity suggested potential transition toward the neural ectodermal lineage (Figure 2A). By day 8, Nanog expression was significantly downregulated, accompanied by a marked reduction in Ki67-positive cells, demonstrating effective suppression of the pluripotency regulatory network. Concurrently, nuclear Sox2 and cytoplasmic Nestin (a neural precursor intermediate filament protein) expression emerged, with morphological transition from compact pluripotent colonies to monolayer neural epithelial-like sheets, confirming successful induction of NSCs (Figure 2B). During mid-differentiation (day 16), RPC specification was evidenced by nuclear expression of Chx10 (a neural retina lineage-specific marker). Complete Sox2 silencing confirmed exit from ectodermal properties and commitment to the neural retina lineage. Ki67-positive cell numbers further decreased, suggesting progressive cell cycle exit, although residual low Ki67 signals in Pax6-positive cells indicated transient proliferative subpopulations within the RPC pool. Therefore, after 16 days of differentiation, the cells had differentiated from ectodermal neural precursor cells to RPCs. Notably, photoreceptor fate determinants CRX and phototransduction protein RCVRN remained undetectable (Figure 2C). By terminal differentiation (day 24), cells expressing nuclear CRX and cytoplasmic RCVRN predominated, marking completion of photoreceptor precursor terminal differentiation. Complete silencing of Pax6, Chx10, and near-undetectable Nestin signals confirmed exit from progenitor states. However, neither rod-specific RHO nor cone-specific L/M Opsin were detected, indicating delayed photopigment expression requiring extended differentiation or additional maturation signals (Figure 2D). The temporal expression patterns of stage-specific markers across the differentiation timeline are summarized in Table 1. Critically, no Müller cells (GS^+^/GFAP^+^), horizontal cells (HC, Calbindin^+^), bipolar cells (BC, PKCα^+^), retinal ganglion cells (RGC, Brn3a^+^), or amacrine cells (AC, GlyT1^+^) were observed at day 24, confirming exclusive photoreceptor lineage specificity and absence of off-target differentiation (Figure 2E; Table 2). The sequential molecular cascade (Nanog→Sox2/Nestin→Chx10→CRX/RCVRN) faithfully recapitulates *in vivo* retinal development,^27^^,^^28^^,^^29^^,^^30^ driven by precise inhibition of WNT signaling pathway.^29^^,^^31^ Nuclear CRX and RCVRN co-expression established molecular competence for phototransduction, while delayed RHO/L/M Opsin expression highlighted the need for post-PPC maturation signals.^32^^,^^33^ The absence of non-photoreceptor markers validated the specificity of this differentiation protocol, providing a high-purity cell source for transplantation studies.

**Table 1:** Different cell types express specific antibodies at various stages as hiPSCs differentiate into PPCs

–	proliferating cell	iPSC	NSC		RPC	photoreceptor		mature photoreceptor	
	Ki67	Nanog	Sox2	Pax6	Chx10	CRX	RCVRN	RHO	L/M Opsin
D0	+	+	+	−					
D8	+	−	+	+	−				
D16	+		−	+	+	−	−		
D24	+			−	−	+	+	−	−

**Table 2:** At the differentiation endpoint, no other retinal neuron types are present besides photoreceptor cells

	Müller		HC	BC	RGC	AC
	GFAP	GS	CALB	PKCα	Brn3a	GLYT1
D24	–	–	–	–	–	–

### *In vivo* OCT evidence of hiPSC-PPC engraftment and retinal microenvironment remodeling

**Figure 3:**
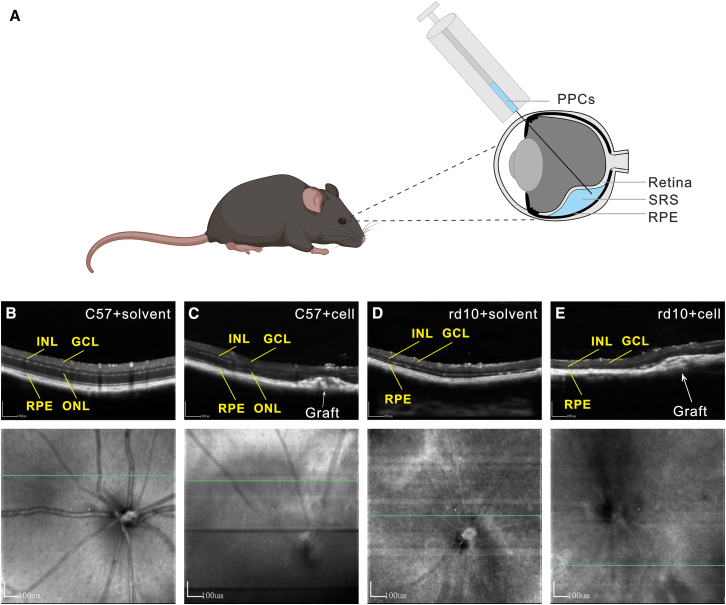
Subretinal injection of hiPSC-derived PPCs in C57 and rd10 mice (A) Schematic of subretinal injection (SRI): Cell suspensions containing hiPSC-derived PPCs were transplanted into the murine SRS. (B) Representative OCT cross-section of sham surgery control (C57 + solvent) demonstrating intact laminar architecture with well-defined GCL, INL, and ONL. (C) OCT imaging of C57 recipient retina 3 months post-transplantation reveals stable subretinal localization of donor cell clusters, with preserved host retinal stratification and no signs of structural disruption. (D) OCT profile of sham surgery control (rd10 + solvent) showing advanced degenerative pathology, including ONL thinning. (E) Longitudinal OCT tracking of rd10 recipients at 90 days post-transplantation shows persistent localization of PPC-derived cellular aggregates in the SRS, with no evidence of retinal detachment. Scale bars represent 100 μm.

To evaluate the retinal structural following transplantation of hiPSC-derived PPCs, we delivered cell suspensions into the SRS of 5-week-old rd10 mice and 8-week-old C57 mice via microinjection. Longitudinal *in vivo* imaging analysis using optical coherence tomography (OCT) was performed at 3 months post-transplantation. A systematic comparison was conducted among four experimental groups: sham surgery controls C57 + solvent, rd10 + solvent, and their respective counterparts receiving cell transplantation (Figure 3A). OCT imaging revealed the formation of localized cellular aggregates in the subretinal compartment post-transplantation. C57 + solvent mice exhibited characteristic retinal laminar organization (Figure 3B), demonstrating uniform thickness across the ganglion cell layer (GCL), inner nuclear layer (INL), and outer nuclear layer (ONL), with continuous integrity of photoreceptor inner segments and outer segments (OS). In contrast, rd10 + solvent mice presented significant degeneration (Figure 3D), manifesting as a reduction in ONL thickness compared to C57 controls. Three-month post-transplantation OCT tracking revealed the persistent presence of donor cell clusters within the SRS in both C57 and rd10 recipients (Figures 3C and 3E), indicating that hiPSC-derived PPCs can be maintained long-term across distinct retinal microenvironments. The observed structural interface between donor cells and host retinal architecture suggests potential anatomical prerequisites for functional restoration.

### Immune-tolerant survival and multidimensional maturation of hiPSC-derived PPCs in murine retinal microenvironments

**Figure 4:**
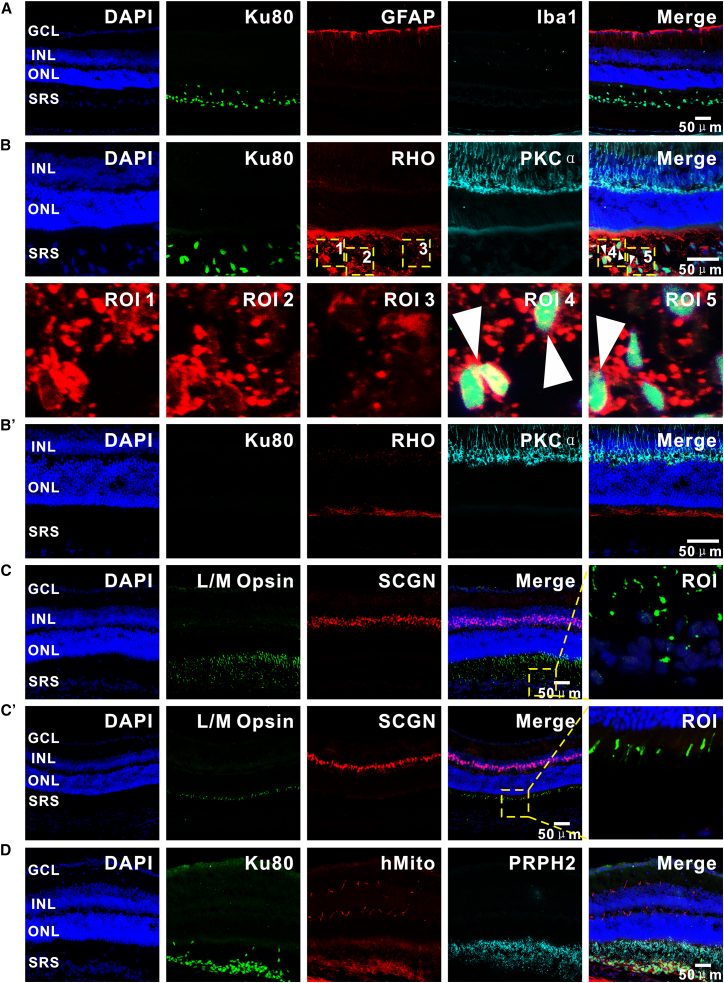
Immunofluorescence characterization of hiPSC-derived PPC survival, differentiation, and ultrastructural maturation in C57 retinas after subretinal transplantation (A) Analysis of glial reactivity and immune response. Astrocyte/activated Müller cell marker GFAP and microglial/macrophage marker Iba1 show virtually undetectable positive signals in the graft, indicating minimal host retinal glial activation and inflammatory immune rejection. (B) Rod photoreceptor differentiation. Robust expression of rod-specific RHO in donor cell clusters suggests commitment to rod lineage. (B′) The absence of detectable RHO immunoreactivity in the SRS distal to the injection site supports antibody specificity. (C) Heterogeneous photoreceptor subtype specification. A subset of donor cells expresses cone-specific L/M Opsin, demonstrating concurrent rod and cone differentiation within the grafted population. Triangular arrows highlight RHO^+^/Ku80^+^ co-localization. (C′) The absence of detectable L/M Opsin immunoreactivity in the SRS distal to the injection site supports antibody specificity. (D) Ultrastructural polarization analysis. Co-localization of PRPH2, a key OS disc assembly protein, with hMito at apical domains of donor cells suggests early-stage formation of light-sensing structural precursors. Scale bars represent 50 μm.

To systematically assess the survival and maturation of hiPSC-derived PPCs following transplantation, retinas from C57 mice were harvested at 3 months post-transplantation (age: about 21 weeks) for comprehensive multidimensional analysis using immunofluorescence staining (Figure 4). Our investigation centered on three critical dimensions: donor cell fate determination, immune microenvironment dynamics, and the establishment of photoreceptor-specific functional architectures. Immunofluorescence analysis revealed minimal glial and immune activation in transplanted retinas. GFAP, a marker for astrocytes and activated Müller cells, exhibited virtually undetectable positive signals near the graft, while microglial/macrophage activation, as indicated by Iba1, remained negligible throughout the retina (Figure 4A). These findings suggest a lack of significant glial reactivity or inflammatory immune rejection toward the xenogeneic grafts, a phenomenon potentially attributable to the immune-privileged nature of the retinal and the intrinsically low immunogenicity of PPCs. Rod differentiation was confirmed by robust expression of RHO within donor cell clusters (Figure 4B). Notably, expression of cone-specific L/M Opsin was observed within donor cell clusters, confirming the differentiation of cones (Figure 4C). HMito further identified metabolically active donor cells at 2 months post-transplantation. Critical insights into ultrastructural maturation emerged from the analysis of PRPH2, a key mediator of OS disc assembly. Polarized localization of PRPH2 at the apical domains of donor cells, co-localizing with hMito signals (Figure 4D), suggested functional initiation of phototransduction machinery assembly, despite the absence of fully laminated OS discs. Collectively, these results demonstrate that grafted PPCs not only retain metabolic vitality and subtype differentiation plasticity but also exhibit progressive ultrastructural specialization at 3 months post-transplantation. The observed immune compatibility between donor cells and host retinal tissue establish a molecular and cellular framework for potential visual function restoration.

### Neuroprotective effects of PPCs in the MNU-induced retinal degeneration model

**Figure 5:**
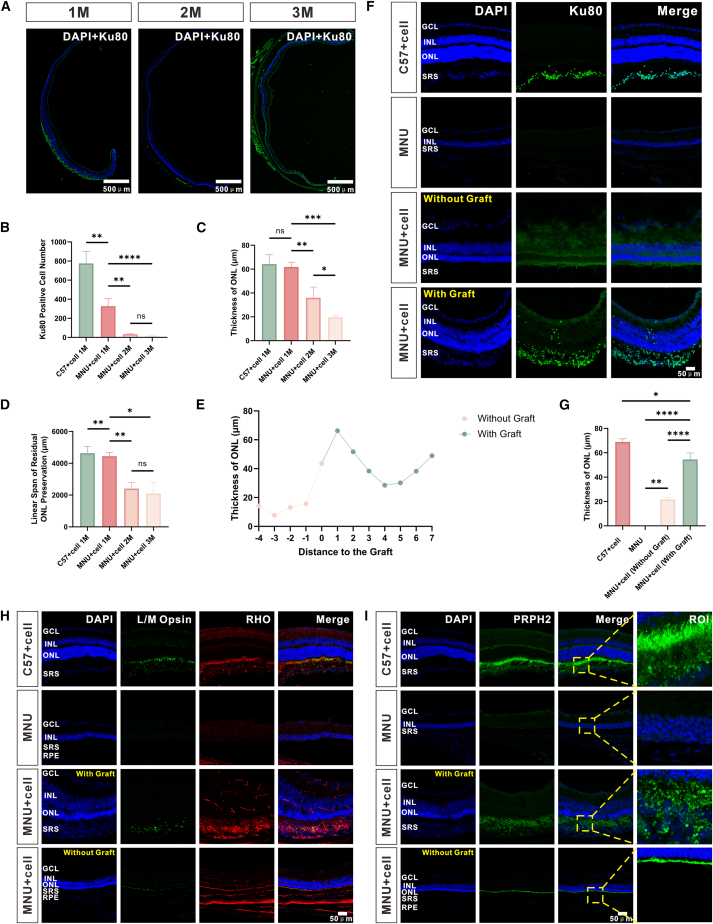
Neuroprotective effects of PPCs transplantation in MNU-induced retinal degeneration in mice (A) Detection of Ku80^+^ human donor cells in the SRS of MNU-induced mice at 1, 2, and 3 months post-transplantation. Scale bars represent 500 μm. (B) Quantification of surviving Ku80^+^ cells in SRS at different time points post-transplantation. (C) Time-dependent changes in ONL thickness. (D) Residual ONL continuous length. Sample sizes for (B)–(D): C57 + cell 1M (*n* = 3), MNU + cell 1M (*n* = 3), 2M (*n* = 4), 3M (*n* = 3). (E) Statistical analysis of ONL thickness across different retinal regions. The origin (*X* = 0) corresponds to the starting point of the transplanted cell cluster. Negative *X* values indicate regions without transplanted clusters, while positive values indicate regions with clusters. *Y* axis represents ONL thickness at each measurement point. Measurements at 500 μm intervals. ONL thickness was significantly greater in regions with transplanted clusters than in those without. (F) DAPI and Ku80 immunofluorescence staining of retinal sections across experimental groups: C57 mice with transplantation (C57 + cell), MNU-modeled control mice without transplantation (MNU), transplanted MNU-modeled mice in regions without cell clusters (MNU + cell, without graft), and transplanted MNU-modeled mice in regions with cell clusters (MNU + cell, with graft). Scale bar represents 50 μm. (G) Quantification of ONL thickness across groups: C57 + cell (healthy control), MNU (untreated degeneration), MNU + cell (without graft; beyond perpendicular projection), and MNU + cell (with graft; perpendicular extension from graft), *n* = 3. Perpendicular projection defined as tissue columns normal to retinal layers extending from graft edges. (H) Expression of rod-specific marker RHO^+^ and cone marker L/M Opsin^+^ in transplanted cell clusters of C57 controls and MNU-modeled mice. Residual OS structures were observed in transplanted retinas but exhibited disorganized arrangement. No RHO^+^ or L/M Opsin^+^ expression was detected in the SRS distal to the injection site of MNU-modeled mice. Scale bar represents 50 μm. (I) PRPH2^+^ (a marker for outer segment disc structures) was detected in both host retinas and transplanted cell clusters of MNU-modeled mice, though spatial organization was disrupted. PRPH2^+^ signals displayed polar alignment in C57 controls but were completely absent in MNU controls. No PRPH2^+^ expression was detected in the SRS distal to the injection site of MNU-modeled mice. Scale bar represents 50 μm. Data are represented as mean ± SEM (one-way ANOVA with appropriate post hoc tests; n.s., not significant, ∗*p* < 0.05, ∗∗*p* < 0.01, ∗∗∗*p* < 0.001, ∗∗∗∗*p* < 0.0001).

To model the progression of RP, eight-week-old C57 mice were intraperitoneally injected with N-methyl-N-nitrosourea (MNU) to establish the MNU-induced retinal degeneration model. On the day of MNU administration, PPCs were transplanted into the SRS of MNU-modeled mice via SRI to evaluate the neuroprotective effects of PPCs throughout RP pathogenesis. Histological examination of MNU-treated mice at 1 month post-transplantation (age: about 9 weeks) revealed complete degeneration of the ONL, while MNU-modeled mice receiving PPCs (MNU + cell) retained significant ONL preservation (Figure 5F). Furthermore, the rod-specific marker RHO and cone marker L/M Opsin were detected in the transplanted cell clusters of MNU + cell mice, but with disorganized morphology compared to the ordered polarity observed in C57 controls. No RHO^+^ or L/M Opsin^+^ expression was detected in the SRS distal to the injection site of MNU + cell mice (Figure 5H). PRPH2, a marker of OS disc structures, was expressed in both the host retina and transplanted cell clusters of MNU + cell mice, but spatial organization was disrupted. In contrast, PRPH2^+^ signals displayed regular polarity in C57 mice and were entirely absent in MNU controls. No PRPH2^+^ expression was detected in the SRS distal to the injection site of MNU + cell mice. Although a thin ONL was partially preserved in these distal regions, the host OS were severely degenerated, appearing collapsed and stacked together as a discontinuous line, consistent with advanced degeneration (Figure 5I).

Regional differences in ONL thickness were quantified relative to PPC graft locations. Regional demarcation was established through immunofluorescence mapping of graft distribution. Ku80^+^ cell clusters within the SRS were precisely bounded by the outermost donor cells at graft peripheries. These edge cells served as fiducial references to define planar partitions perpendicular to the retinal layers. Retinal tissue extending perpendicularly from graft boundaries through all retinal layers was classified as “with graft” regions, while regions beyond this perpendicular projection comprised “without graft” regions. The latter designation required histological confirmation of complete absence of Ku80^+^ nuclei throughout all retinal layers in these zones. At sites of “with graft,” ONL thickness measured 43.18 μm (43.18 ± 5.06 μm), significantly greater than “without graft” (12.70 ± 1.73 μm; *p* < 0.01) (Figure 5E), confirming distance-dependent neuroprotection. Notably, despite reduced ONL thickness in cluster-free regions compared to cluster-adjacent regions, these regions still retained partial protection, as age-matched MNU control mice exhibited complete ONL loss (0 ± 0 μm; *p* < 0.01 vs. MNU + cell, without graft: 21.54 ± 0.82 μm) (Figure 5F), implying indirect paracrine-mediated neuroprotection. However, direct cell-contact regions (MNU + cell, with graft) demonstrated superior ONL preservation (54.47 ± 3.09 μm; *p* < 0.0001 vs. MNU + cell, without graft). Despite significant ONL protection, the ONL thickness in MNU + cell mice remained lower than in healthy C57 + cell controls (68.89 ± 1.47 μm; *p* < 0.05) (Figure 5G).

To assess the long-term neuroprotective effects of PPCs, longitudinal analysis was performed in MNU-modeled mice over 1–3 months post-transplantation (age: about 9–21 weeks; Figures 5G and 5H). Quantitative analysis of surviving Ku80^+^ donor cells in the SRS revealed that the MNU + cell 1M group retained 327 ± 46 cells, significantly fewer than C57 + cell 1M controls (775 ± 71 cells; *p* < 0.001). Donor cell survival progressively declined in MNU-modeled mice, dropping to 34 ± 5 cells at 2 months (MNU + cell 2M; *p* < 0.01 vs. 1M) and 12 ± 3 cells at 3 months (MNU + cell 3M; *p* < 0.01 vs. 2M), indicating time-dependent exacerbation of donor cell loss in the pathological microenvironment (Figure 5B). Concurrently, ONL thickness decreased from 61.84 ± 2.25 μm in the MNU + cell 1M group to 35.97 ± 4.51 μm at 2 months (*p* < 0.01 vs. 1M) and 19.56 ± 0.73 μm at 3 months (*p* < 0.05 vs. 2M), reflecting diminishing neuroprotective efficacy over time (Figure 5C). ONL continuity spanning was defined as the maximal linear distance of continuous ONL preservation along the retinal plane. Residual ONL continuity also declined significantly, with continuous ONL distribution lengths measuring 4,444 ± 134 μm at 1M, 2,408 ± 193 μm at 2M (*p* < 0.01 vs. 1M), and 2,087 ± 415 μm at 3M (*p* < 0.001 vs. 1M), indicating progressive spatial contraction of neuroprotection (Figure 5D).

Comparative analysis of SRI and intravitreal injection (IVI) strategies in the MNU model revealed distinct neuroprotective outcomes. At 1 month post-transplantation, immunofluorescence quantification of ONL thickness and photoreceptor distribution (Figure S2B) demonstrated that the SRI group exhibited significantly greater average ONL thickness (61.84 ± 2.25 μm) than the IVI group (36.02 ± 2.12 μm; *p* < 0.01) (Figure S2C). Spatial distribution analysis further confirmed broader photoreceptor preservation in the SRI group, with residual ONL continuity spanning 4,444 ± 134 μm, compared to 2,211 ± 246 μm in the IVI group (*p* < 0.01) (Figure S2D). These results collectively validate that SRI achieves superior neuroprotective efficacy in the MNU model, with enhanced protection intensity and spatial coverage relative to IVI.

### Photoreceptor maturation and synaptic reconnection of hiPSC-PPCs in degenerative retinal SRS

**Figure 6:**
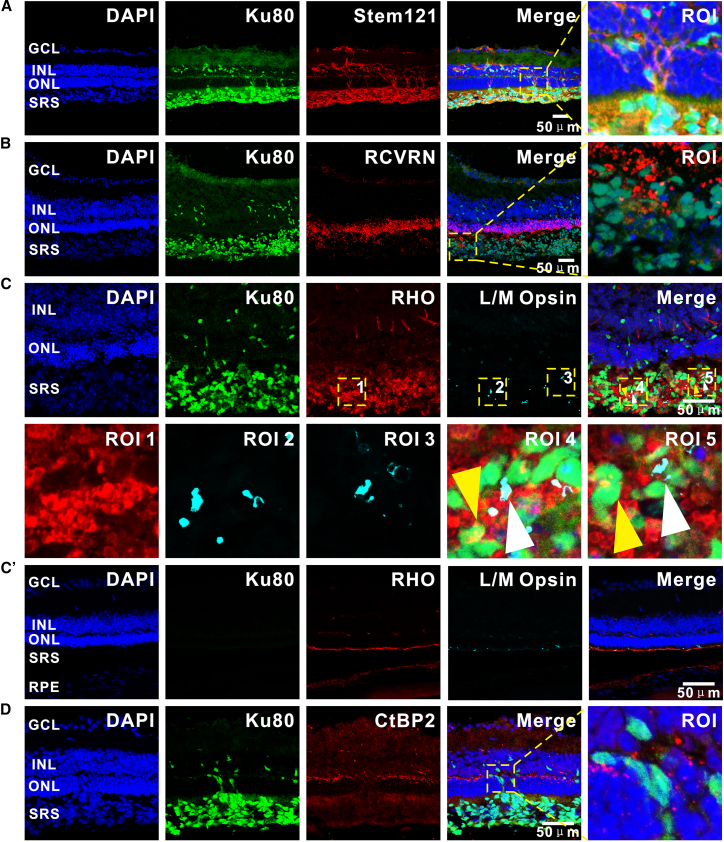
Photoreceptor lineage commitment and presynaptic marker expression of hiPSC-derived PPCs in MNU-induced retinal degeneration (A) Human donor cell survival: schematic of human-specific marker validation. Ku80^+^/STEM121^+^ donor cells were observed in the host ONL and SRS. (B) Photoreceptor specification and phototransduction initiation: subretinal grafts exhibited expression of the mature photoreceptor marker RCVRN in Ku80^+^ donor cells. (C) Subtype-specific maturation: Rod/cone differentiation was confirmed by RHO expression (rod-specific) and L/M Opsin expression (cone-specific) in Ku80^+^ clusters of donor subsets, with merged imaging demonstrating dual lineage commitment. Yellow arrows highlight RHO^+^/Ku80^+^ co-localization. White arrows highlight L/M Opsin^+^/Ku80^+^ co-localization. (C′) No RHO^+^ or L/M Opsin^+^ expression was detected in the SRS distal to the injection site of MNU-modeled mice. (D) Presynaptic marker expression: CtBP2^+^ puncta were observed near Ku80^+^ donor cells. Scale bars represent 50 μm.

HiPSC-derived PPCs transplanted into 8-week-old MNU-modeled mice exhibited robust photoreceptor lineage differentiation (Figure 6). Dual labeling with human-specific nuclear marker Ku80 and cytoplasmic marker STEM121 confirmed the human origin and sustained metabolic viability of transplanted PPCs at 1 month post-transplantation (age: about 12 weeks), with preserved structural integrity. Ku80^+^/STEM121^+^ donor cells were observed in the host ONL and SRS (Figure 6A). It should be noted that donor-cell localization within the ONL may reflect leakage or misplacement during injection. This is consistent with the finding that in SRI surgery, substantial reflux into the vitreous and off-target distribution of cells are common phenomena.^34^ Donor cells showed strong expression of the phototransduction effector protein RCVRN (Figure 6B), indicating not only fate commitment to photoreceptor lineages but also initiation of molecular machinery assembly for phototransduction. Subtype analysis further revealed co-expression of rod-specific RHO and cone-specific L/M Opsin within the grafted cell population (Figure 6C), suggesting that the degenerative retinal microenvironment supports functional maturation of donor cells. Furthermore, immunofluorescence analysis revealed the expression of the presynaptic marker CtBP2 within donor clusters. These CtBP2^+^ puncta were observed in close apposition to Ku80^+^ donor cells (Figure 6D). This presynaptic specialization resembles the morphology of synaptic ribbons in native photoreceptors. However, functional synaptic transmission remains to be demonstrated.

**Figure 7:**
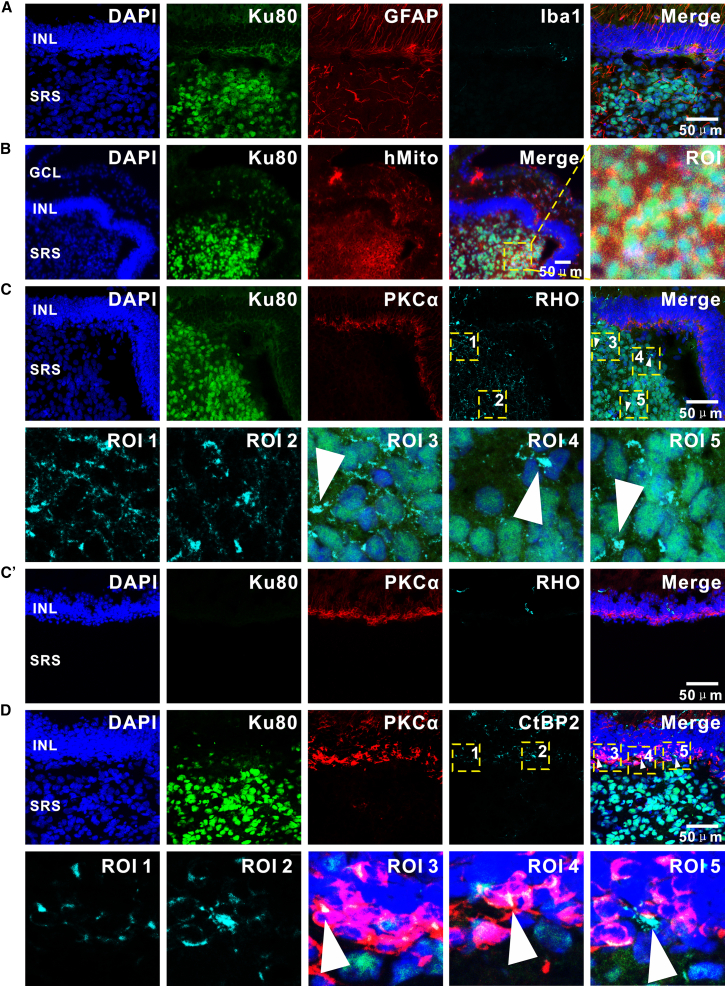
Photoreceptor lineage commitment and presynaptic structure formation of hiPSC-derived PPCs in rd10 retinas (A) Glial and immune microenvironment characterization: analysis of activated Müller cell marker GFAP reveals localized Müller cell activation near the graft. (B) Metabolic viability assessment: hMito confirms abundant metabolically active donor cells within the grafted clusters. (C) Photoreceptor maturation: Robust expression of rod-specific RHO in donor cells demonstrates subtype-specific differentiation. Triangular arrows highlight RHO^+^/Ku80^+^ co-localization. (C′) No RHO^+^ expression was detected in the SRS distal to the injection site of rd10 mice. (D) Immunostaining for presynaptic marker CtBP2 and BC marker PKCα shows CtBP2^+^ puncta located near PKCα^+^ BC dendrites. Triangular arrows highlight areas of proximity. Scale bars represent 50 μm.

In rd10 murine retinas analyzed at 3 months post-transplantation (age: about 18 weeks), hiPSC-derived PPCs were observed to persist and exhibit specific structural characteristics within the degenerative microenvironment (Figure 7). There is evident Müller cell activation near the graft, characterized by increased GFAP^+^ fibers (Figure 7A). Despite these pathological responses, hMito confirmed persistent metabolic activity in donor clusters (Figure 7B). Robust RHO expression validated photoreceptor maturation of PPCs within rd10 retinas (Figure 7C). Notably, discrete CtBP2^+^ presynaptic puncta within donor cells were observed in close proximity to host BC dendrites (Figure 7D). These structural observations suggest that the degenerative microenvironment can permit donor cell persistence and the formation of presynaptic-like specializations.

To evaluate functional outcomes in the degenerative context of the rd10 model, we performed transmission electron microscopy (TEM) of photoreceptor synapses, flash visual evoked potentials (FVEP), and behavioral assays including the light/dark box test and optomotor response (OMR). In transplanted rd10 mice, EM revealed presynaptic ribbon synapses but no clear postsynaptic partners (Figure S3B). At three months post-transplantation, FVEP recordings did not show significant improvement, likely due to the limited number of donor cells and incomplete re-establishment of neural circuits (Figures S3C and S3D). Similarly, behavioral tests showed positive trends that did not reach statistical significance (Figures S3E and S3F). Although the functional outcomes were modest, these comprehensive analyses provide valuable insights into the challenges of achieving synaptic connectivity in advanced degeneration and will help to guide future optimization strategies.

## Discussion

Our study establishes an RA-free chemically induction system for high-efficiency PPC production from human iPSCs, demonstrating robust therapeutic efficacy through functional integration and microenvironment-guided maturation in retinal degeneration models. Retinal degenerative pathologies, particularly RP and AMD, remain clinically intractable due to the irreversible loss of photoreceptors and the inherently limited regenerative capacity of retinal tissue. Leveraging a synchronized pharmacological modulation strategy, we established a 24-day hierarchical differentiation protocol that transitions human-induced pluripotent stem cells through sequential developmental stages: NSC commitment, RPC maturation, and ultimately PPC generation, achieving a 50% temporal compression compared to standard organoid differentiation paradigms.^35^^,^^36^ This platform yielded CRX^+^/LHX4^+^ PPCs with >99.9% purity, surpassing 3D retinal organoid efficiency while obviating genetic manipulation or exogenous transcriptional regulators,^37^ thereby addressing critical safety barriers for clinical translation.

Stage-resolved molecular profiling delineated key retinogenic developmental trajectories, beginning with epigenetic Nanog repression, progressing through Sox2/Nestin co-activation-mediated neural induction, advancing to Pax6/Chx10 co-expression-driven retinal specification, and ultimately achieving CRX/RCVRN transcriptional activation marking photoreceptor terminal differentiation.^38^^,^^39^ The delayed onset of RHO/L/M Opsin expression, which becomes detectable only following transplantation, recapitulates the cell-autonomous developmental timeline of photoreceptors, suggesting that photoreceptor subtype commitment is contingent upon extrinsic tissue microenvironmental signals.^40^^,^^41^ Notably, the complete absence of non-photoreceptor lineages (Müller/HC/BC/RGC/AC) validates unparalleled lineage precision in chemically guided differentiation.^42^

To validate clinical translatability, hiPSC-derived PPCs were transplanted into two established retinal degeneration models (rd10 mutants and MNU-induced mice) under immunosuppression (Cyclosporine A). Longitudinal OCT showed the persistent presence of donor cell clusters for ≥3 months within the SRS. Postmortem immunohistochemistry revealed terminal differentiation into rod- and cone-like lineages, evidenced by RHO^+^/L/M Opsin^+^ expression and phototransduction complex assembly.^43^ Donor clusters exhibited polarized PRPH2^+^ aggregates mimicking native photoreceptor OS architecture, though lamellar disc organization remained immature. This spatial recapitulation of photoreceptor ultrastructure implies functional competency for phototransduction initiation,^44^ while CtBP2^+^ puncta (presynaptic specializations) adjacent to host BC dendrites were observed.^45^ Whether these structures represent functional synapses requires further investigation. Successful synaptic integration between grafted PPCs and host BCs depends on a synergy of cell-intrinsic mechanisms within the donor cells and host environmental support, including chemotactic guidance and activity-dependent refinement.^46^^,^^47^^,^^48^ However, in the context of the present study, several interconnected factors likely limited the functional efficacy of these nascent connections. The insufficient number of integrated donor cells likely resulted in a synaptic density below the threshold required for network-level signal amplification and detectable functional rescue. Furthermore, the prescribed 3-month observation window, while sufficient for structural presynaptic formation, may be insufficient for the complete maturation, stabilization, and activity-dependent refinement of graft-host synaptic circuits. This challenge is exacerbated by the degenerative rd10 microenvironment, where host BC dendritic retraction and Müller glia-mediated gliosis present formidable anatomical and biochemical barriers to optimal synaptic pairing. Consequently, while presynaptic ribbons were observed, the formation of functionally robust and numerous synaptic triads was likely impeded. This mechanistic interpretation is consistent with our electrophysiological and behavioral data, which showed positive but statistically non-significant trends toward functional improvement. Nevertheless, electrophysiological confirmation of signal transduction through retinal circuits remains a critical next-step validation.^49^^,^^50^^,^^51^^,^^52^

In this study, we established an MNU-induced retinal degeneration model in WT mice via intraperitoneal injection of MNU, mimicking the progressive pathogenesis of RP. MNU administration triggered gradual apoptosis of photoreceptor cells.^53^ On the day of MNU induction, PPCs were transplanted into the SRS of MNU-modeled mice via SRI, allowing evaluation of the neuroprotective effects of PPCs throughout RP progression. Notably, significant regional differences in ONL thickness were observed within the same retina, with regions directly adjacent to transplanted cell clusters exhibiting superior structural preservation, suggesting localized neuroprotection mediated by cell-cell contact or paracrine signaling. Longitudinal analyses revealed a progressive decline in donor cell survival over time, with fewer surviving donor cells in the SRS of MNU-modeled mice compared to age-matched healthy C57 mice. This implies that donor cell survival may depend on a healthy retinal microenvironment, and the pathological milieu likely accelerates donor cell loss. Concurrently, preserved ONL thickness and residual ONL continuity diminished over time, indicating a gradual decline in both the intensity and spatial coverage of neuroprotection. These dual-dimension attenuations (temporal and spatial) may result from limited donor cell survival, dynamic deterioration of the pathological microenvironment, or reduced diffusion efficiency of neurotrophic factors due to local gradients of cytotoxic mediators or declining trophic support.

The vitreous body, a transparent gel-like structure primarily composed of water, collagen, and hyaluronic acid, exhibits extremely low fluidity in mammals, which may be further reduced under pathological conditions such as inflammation or degeneration. Its high viscosity and structural complexity restrict free cellular diffusion. Cells delivered via IVI are often trapped within the vitreous fibrous network, aggregating near the injection site and failing to distribute uniformly. Even if neurotrophic factors are secreted by these cells, their diffusion is constrained by vitreous viscosity, retinal laminar architecture, and enzymatic degradation, typically forming localized concentration gradients (effective range: ∼1–2 mm). Consequently, distal retinal regions remain inadequately protected due to insufficient factor concentrations. This explains the limited spatial protection observed in MNU-modeled mice following IVI of PPCs, where neuroprotection failed to extend across the entire retina. In contrast, SRI enables direct contact between donor cells and the photoreceptor layer, with cells distributing evenly within the SRS and paracrine factors diffusing more efficiently. Accordingly, SRI outperformed IVI in the MNU model, achieving significantly enhanced neuroprotective efficacy and broader spatial coverage. Previous studies have shown that intravitreally delivered stem/progenitor cells primarily exert protective effects on adjacent regions (e.g., retinal ganglion cell layer or inner retinal layers) but exhibit limited efficacy in distal photoreceptor layers.^54^ The inner limiting membrane, situated between the vitreous and retina and composed of a basement membrane and glial cell processes, features nanopores that physically restrict cellular migration to outer retinal layers. Furthermore, intraocular fluid primarily flows anteriorly from the vitreous cavity to the anterior chamber, exiting via aqueous humor circulation. This pathway limits direct interaction with outer retinal layers, potentially hindering effective delivery of cells or factors to the ONL. The absence of donor cells in the ONL following IVI may thus be attributed to the ILM barrier and the directional constraints of intraocular fluid dynamics.

From a translational medicine perspective, our observations indicate that hiPSC-PPCs can achieve long-term persistence in the host SRS under systemic immunosuppression, which merits further investigation into their clinical translational potential.^55^^,^^56^^,^^57^^,^^58^ This study further reveals that although PPCs at the terminal differentiation stage are not fully mature, their progressive post-transplant expression of opsins highlights the capacity of the host microenvironment to drive terminal maturation of donor cells.^59^ The distinct engraftment patterns of donor cells in degenerative versus healthy retinas highlight the necessity for stage-adapted personalized therapeutic strategies: early interventions may prioritize leveraging the neuroplastic potential of the host microenvironment, while advanced-stage therapies require combinatorial stromal modulation to establish permissive integration conditions. This necessitates urgent optimization of transplantation protocols through combinatorial approaches such as adjuvant neuroprotective interventions or co-delivery of RPE cellular components to ensure persistent graft viability and synaptic integrity in the host retinal circuitry.^46^^,^^60^^,^^61^ Our analyses reveal that donor cell attrition occurs independently of classical neuroinflammation, as evidenced by absent microglial activation (Iba1^−^) across both models and timepoints (Figures 4A and 7A). Instead, non-inflammatory mechanisms dominate: (1) surgical and adaptive stresses likely trigger apoptosis in early phases (Figure 5B); (2) trophic deficiencies in degenerative microenvironments limit long-term support, evidenced by neuroprotection gradients in MNU retinas (Figure 5E); and (3) chronic gliosis in rd10 creates hostile integration niches (Figure 7A). The accelerated cell loss in degenerative versus healthy microenvironments (Figure 5B) underscores the critical role of host tissue integrity. Future therapies should target these pathways through combinatorial trophic support and anti-apoptotic strategies. Furthermore, inherent interspecies discrepancies between animal models and human diseases, particularly manifested in parameters such as disease progression kinetics, retinal laminar architecture, immune response thresholds, and immunosuppressive regimen translatability, therefore necessitate rigorous validation of therapeutic generalizability in non-human primate models during preclinical development.^11^ It is also important to acknowledge that the current study has limitations in functional validation and translational generalizability, which are discussed in detail in the “limitations of the study” section.

In conclusion, human iPSC-derived PPCs generated via a serum-free chemical protocol demonstrate therapeutic potential across distinct retinal degeneration models. In the genetic rd10 model, transplanted PPCs exhibited structural maturation and formed presynaptic specializations, though full synaptic triads were not identified. In acute MNU-induced injury, early PPC intervention showed neuroprotective effects, attenuating photoreceptor loss and preserving retinal architecture. Analysis of the rd10 model indicated that donor cells expressed presynaptic proteins (e.g., CtBP2) and were observed in close apposition to host bipolar neurons. This configuration could potentially permit synaptic communication within a permissive microenvironment. However, the absence of significant functional recovery in cortical and behavioral assays suggests that such structural proximity alone may be insufficient. Therefore, future therapeutic strategies should combine approaches to enhance graft longevity with methods to promote functional connectivity. This work establishes a foundation for cell replacement therapy in early-stage degeneration but highlights the necessity to define optimal intervention windows and microenvironmental modifiers for clinical translation. Although our study did not yield statistically significant functional recovery, the combined use of ultrastructural, electrophysiological, and behavioral assessments provides a valuable reference framework for evaluating synaptic integration in advanced retinal degeneration. By systematically applying these complementary approaches, we highlight both the potential and the limitations of current strategies, thereby offering insights that may inform future experimental design and therapeutic development in the field.

### Limitations of the study

The limitations inherent in this experimental framework define imperative research trajectories for subsequent investigations. First, the precise mechanism underlying donor cell localization within the ONL cannot be definitively determined from our current data. Without real-time longitudinal imaging, we cannot distinguish between the relative contributions of active migration and passive displacement. Passive displacement during the injection procedure represents a plausible alternative explanation for this observation among others. Even with standardized injection techniques, reflux of cells into the vitreous and dispersion within the SRS are common obstacles during SRI. Substantial leakage of cells back into the vitreous has been observed during SRI procedures, reducing the number of cells in the intended location and potentially leading to off-target distribution.^34^ This well-documented phenomenon of off-target distribution provides a plausible technical explanation for our observation. Therefore, the Ku80^+^ cells we observed in the ONL may partially reflect such injection-related misplacement rather than active migration or integration. Second, we acknowledge the limited functional validation of synaptic circuits. Although ultrastructural evidence of presynaptic ribbons was observed, the lack of electrophysiological corroboration (e.g., via rod BC patch-clamp or dynamic calcium imaging) precludes conclusive verification of functional synaptic transmission within reconstructed networks. Third, the metabolic coupling mechanisms between donor cells and the RPE remain unresolved, and pre-existing RPE deficits may critically compromise long-term photoreceptor survival.^62^^,^^63^ Finally, the evaluation of visual function was restricted to basic light response and optomotor reflexes. The absence of more complex assessments (e.g., shape discrimination acuity and motion detection) limits the holistic evaluation of the intervention’s potential to restore neurologically integrated visual pathways, thereby constraining the translational generalizability of our findings.

Future investigations should prioritize longitudinal *in vivo* imaging through repetitive non-invasive modalities such as adaptive optics scanning laser ophthalmoscopy or *in vivo* two-photon microscopy to dynamically track donor cell fate and distribution, which would definitively elucidate the mechanisms behind their localization. Additionally, optogenetic interrogation of graft activity, development of human retinal organoid transplantation models, and integrative multi-omics profiling of host-graft interaction networks should be pursued to systematically refine therapeutic strategies and elucidate their mechanistic underpinnings.

## Resource availability

### Lead contact

Further information and requests for resources and reagents should be directed to and will be fulfilled by the lead contact, Yin Shen (yinshen@whu.edu.cn).

### Materials availability

All unique/stable reagents generated in this study are available from the lead contact with a completed Materials Transfer Agreement.

### Data and code availability

• The raw scRNA-seq and bulk RNA-seq data reported in this paper have been deposited in the Genome Sequence Archive (GSA)^64^ in National Genomics Data Center,^65^ China National Center for Bioinformation/Beijing Institute of Genomics, Chinese Academy of Sciences, under accession number GSA: HRA013729. Processed scRNA-seq and bulk RNA-seq data have been deposited in OMIX under accession numbers OMIX: OMIX012320 and OMIX: OMIX012264, respectively.
• This paper does not report original code.
• Any additional information required to reanalyze the data reported in this paper is available from the lead contact upon request.

## Acknowledgments

This work was supported by grant from the 10.13039/100014718National Natural Science Foundation of China (no. 82471086). We thank Dr. Nanxiang Xiong (Union Hospital, Tongji Medical College, Huazhong University of Science and Technology) for providing the hiPSC line.

## Author contributions

Y.S. and Y.D. designed the study. L.N. and J.W. generated the hiPSCs and developed the protocol for 2D differentiation culture of PPCs. Y.D., J.C. and X.Y. conducted IHC and TEM analysis. Y.D. and Y.L. conducted transplantation surgeries. Y.D. and X.Y. conducted FVEP, OMR and light/dark box test. J.Z. and J.W. conducted single-cell and bulk RNA sequencing analysis. Y.D. and G.T. conducted quantitative analysis. Y.D., J.W. and Y.S. wrote the manuscript. All authors reviewed and approved the final manuscript.

## Declaration of interests

L.N. and J.Z. are employees of iRegene Therapeutics Co., Ltd. J.W. is a founder of iRegene Therapeutics Co., Ltd. J.W. and L.N. are inventors on the issued Chinese patent CN 112608883 B, which covers methods for generating hiPSC-derived PPCs.

## STAR★Methods

### Key resources table

**Table:** 

REAGENT or RESOURCE	SOURCE	IDENTIFIER
**Antibodies**		

Mouse anti-Stem121	TAKARA	Cat# Y40410; RRID:AB_2801314
Goat anti-Ku80	R&D Systems	Cat# AF5619; RRID:AB_2218619
Rabbit anti-Nanog	CST	Cat# 4903S
Rabbit anti-Sox2	R&D Systems	Cat# AF2018; RRID:AB_355110
Rabbit anti-Ki67	Invitrogen	Cat# MA5-14520; RRID:AB_10979488
Rabbit anti-Pax6	Sigma-Aldrich	Cat# ab2237; RRID:AB_1587367
Rabbit anti-Nestin	Sigma-Aldrich	Cat# SAB4200347
Sheep anti-Chx10	Exalpha	Cat# X1180P; RRID:AB_2314191
Mouse anti-CRX	Novus	Cat# H00001406-M02; RRID:AB_538009
Rabbit anti-Recoverin (RCVRN)	Sigma-Aldrich	Cat# AB5585; RRID:AB_2253622
Mouse anti-Rhodopsin (RHO)	Sigma-Aldrich	Cat# MAB5316; RRID:AB_2156055
Rabbit anti-L/M Opsin	Sigma-Aldrich	Cat# AB5405; RRID:AB_177456
Rat anti-Glial Fibrillary Protein (GFAP)	Sigma-Aldrich	Cat# 345860; RRID:AB_10685458
Mouse anti-Glutamine Synthetase (GS)	BD Biosciences	Cat# 610517; RRID:AB_397879
Rabbit anti-Calbindin (CALB)	SWANT	Cat# CB38; RRID:AB_10000340
Rabbit anti-PKCα	SANTA CRUZ	Cat# sc-208; RRID:AB_2168668
Rabbit anti-Brn3a	Sigma-Aldrich	Cat# MAB1585; RRID:AB_94166
Rabbit anti-GLYT1	Novus	Cat# NBP1-81820; RRID:AB_11039216
Mouse anti-Iba1	Wako	Cat# 012-26723
Sheep anti-Secretagogin (SCGN)	Biovendor	Cat# RD184120100; RRID:AB_2034062
Rabbit anti-Peripherin 2 (PRPH2)	Proteintech	Cat# 18109-1-AP; RRID:AB_10665364
Mouse anti-Human Mitochondrial Antigen (hMito)	Abcam	Cat# ab92824; RRID:AB_10562769
Mouse anti-CtBP2	BD Biosciences	Cat# 612044; RRID:AB_399431
Alexa Fluor® 488 AffiniPure Donkey Anti-Rabbit IgG (H + L)	Jackson	Cat# 711-545-152; RRID:AB_2313584
Alexa Fluor® 647 AffiniPure Donkey Anti-Rabbit IgG (H + L)	Jackson	Cat# 777-605-152
Alexa Fluor® 488 AffiniPure Donkey Anti-Goat IgG (H + L)	Jackson	Cat# 705-545-147; RRID:AB_2336933
Alexa Fluor® 488 AffiniPure Donkey Anti-Mouse IgG (H + L)	Jackson	Cat# 717-545-150
Alexa Fluor® 594 AffiniPure Donkey Anti-Mouse IgG (H + L)	Jackson	Cat# 715-585-151; RRID:AB_2340855
Alexa Fluor® 594 AffiniPure Donkey Anti-Sheep IgG (H + L)	Jackson	Cat# 713-585-147; RRID:AB_2340748
Alexa Fluor® 488 AffiniPure Donkey Anti-Rat IgG (H + L)	Jackson	Cat# 712-545-150; RRID:AB_2340683

**Chemicals, peptides, and recombinant proteins**		

Cyclosporine A Soft Capsules	Hangzhou Zhongmei Huadong Pharmaceuticals	Cat# H10960122
mTeSR1 Medium	STEMCELL Technologies	Cat# 85850
Matrigel	Corning	Cat# 356231
Dulbecco’s Phosphate-Buffered Saline (DPBS)	Gibco	Cat# C14190500BT
Dimethyl Sulfoxide (DMSO)	Sigma-Aldrich	Cat# D2650
Laminin 521	Corning	Cat# 354221
AM580	Selleck	Cat# S2933
Dulbecco’s Modified Eagle Medium (DMEM)	Gibco	Cat# C11995500BT
Y-27632 HCl	Selleck	Cat# S1049
DAPI	Servicebio	Cat# G1012
Antifade Mounting Medium	Servicebio	Cat# G1401
Paraformaldehyde (PFA)	Hushi	Cat# 800966618
Phosphate Buffer Saline (PBS)	Servicebio	Cat# G0002
Bovine Serum Albumin (BSA)	Biofroxx	Cat# 143184
Triton X-100 (TRI)	Sigma-Aldrich	Cat# 93289
Ethylenediaminetetraacetic Acid	Servicebio	Cat# 174621
Vitrification Solution	Youkang Biotechnology	Cat# NC1011
Compound Tropicamide Eye Drops	DiRui Pharmaceuticals	Cat# H20103127
Proparacaine Hydrochloride Eye Drops	ALCON-COUVREUR	Cat# H20160133
Isoflurane	RWD Life Science	Cat# R510-22-10
NouvNeu™ Neural Induction Medium	iRegene Therapeutics	N/A
NouvNeu^TM^ Neural Differentiation Medium	iRegene Therapeutics	N/A
1% Carboxymethylcellulose Drops	ALLERGAN	Cat# H20140946
Tobramycin and Dexamethasone Eye Ointment	SIEGFRIED EL MASNOU	Cat# HJ20181126
Trypan Blue	Solarbio	Cat# C0040
0.5% Carboxymethylcellulose Drops	ALLERGAN	Cat# H20140945
OCT Compound	SAKURA	Cat# 4583
N-Methyl-N-Nitrosourea (MNU)	Aladdin	Cat# N136701
Sucrose	Hushi	Cat# 10021418
Glutaraldehyde	Absin	Cat# abs9277
Osmium Tetroxide	SPI	Cat# T99863
Ethanol	Sinopharm Chemical Reagent	Cat# 10009218
Acetone	Sinopharm Chemical Reagent	Cat# 10000418
SPI-PonTM 812 Epoxy Embedding Kit	SPI	Cat# SPI-02660
Uranyl Acetate	SPI	Cat# T99866
Lead Nitrate	Sinopharm Chemical Reagent	Cat# 10099-74-8
Trisodium Citrate	Sinopharm Chemical Reagent	Cat# 10019418

**Deposited data**		

Raw RNA-seq data	This paper	HRA013729
Processed scRNA-seq data	This paper	OMIX012320
Processed bulk RNA-seq data	This paper	OMIX012264

**Experimental models: Cell lines**		

Human: Passage 20 iPS cells	Huazhong University of Science and Technology	N/A

**Experimental models: Organisms/strains**		

C57BL/6J mice	Zikeheng Biotechnology	RRID:IMSR_JAX:000664
Pde6b^rd10/rd10^ mice	Cyagen Biosciences	Cat# C001276

**Software and algorithms**		

Adobe Photoshop CS5	Adobe Systems Inc	https://www.adobe.com/
ZEN Black	Carl Zeiss AG	https://www.zeiss.com/microscopy/int/products/microscope-software/zen.html
LAS X	Leica Application Suite X	https://www.leica microsystems.com/products/microscope software/p/leica-las-x-ls/
GraphPad Prism 8.0	GraphPad Software Inc	http://www.graphpad.com/scientific-software/prism/
MATLAB	The MathWorks Inc	http://ch.mathworks.com/products/matlab.html

### Experimental model and study participant details

#### Animals

The experimental animals used in this study included male *C57BL/6J* (C57) WT mice (8 weeks old) purchased from Wuhan Zikeheng Biotechnology Co., Ltd., and male *Pde6b*^*rd10/rd10*^ (rd10) mice (5 weeks old) obtained from Cyagen Biosciences Inc. Male mice were exclusively used in this study based on the established understanding that retinal degeneration models like rd10 and MNU-induced degeneration exhibit comparable progression rates and outcomes between sexes, and to minimize experimental variability introduced by the estrous cycle. All mice were housed in a specific pathogen-free-grade barrier facility at the Animal Experiment Center of Wuhan University Medical Research Institute, with environmental conditions maintained at 22°C–26°C, 40–70% humidity, and a 12-h light/dark cycle. Mice were provided gamma-irradiated sterile feed and purified water *ad libitum*. All animal experiments and procedures were approved by the Animal Ethics Committee of Wuhan University (Approval No: MRI2024-LAC010) and strictly followed the NIH Guide for the Care and Use of Laboratory Animals. All personnel involved in animal handling had obtained certification in relevant technical procedures. All transplantation recipients received systemic immunosuppression with Cyclosporine A (Hangzhou Zhongmei Huadong Pharmaceutical Co., Ltd., China). Cyclosporine A soft capsules were dissolved in sterile saline (2 mg/mL) and administered via oral gavage at 10 mg/kg body weight. Treatment commenced 48 h prior to transplantation and continued every other day for the first week, followed by weekly maintenance dosing until the experimental endpoint. This regimen effectively minimized xenograft rejection while maintaining animal health.

#### Cell line

The hiPSC line used in this study was generated from de-identified cord blood of a healthy 28-year-old female donor of Chinese nationality (ethnicity not specified). Sample collection and hiPSC derivation were performed with written informed consent and approval by the Ethics Committee of Tongji Medical College, Huazhong University of Science and Technology (protocol no. [2019] S1213). The cell line was rigorously characterized, which included authentication by short tandem repeat (STR) profiling, a normal karyotype, positive expression of pluripotency markers, and demonstrated trilineage differentiation potential. Additionally, the cells were consistently confirmed to be free of mycoplasma contamination and human viral pathogens, and maintained sterility post-thaw, during expansion, and throughout differentiation.

### Method details

#### Maintenance of hiPSC culture

HiPSCs were maintained in T25 flasks pre-coated with Matrigel and cultured in fresh mTeSR1 maintenance medium (STEMCELL Technologies Inc., Canada). Cells were passaged at 70–80% confluency using 0.05% EDTA dissociation solution (Servicebio Technology Co., Ltd, China). Cells were then resuspended in fresh mTeSR1 maintenance medium, and subcultured at a 1:10 split ratio into freshly prepared Matrigel-coated flasks. 10 μM Rock inhibitor Y-27632 (Selleck Chemicals LLC, USA) was added on the first day after passaging to facilitate cell attachment.

#### Generation of PPCs from hiPSCs via small molecule-driven specification

HiPSCs were grown in Matrigel (Corning Incorporated, USA)-coated 6-well plates until 80–90% confluent, followed by dissociation with Accutase. Cells were then seeded at a density of 3.5–4.0×10^6^ cells/well on Laminin 521 (Corning Incorporated, USA)-coated plates (5 μg/mL) in NouvNeu Neural Induction Medium (iRegene Therapeutics Co., Ltd, China) to initiate differentiation. Neural induction was performed using NouvNeu Neural Induction Medium for neural stem cell specification from Day 0 to Day 10.^66^ RPC specification was then achieved using NouvNeu Neural Differentiation Medium (iRegene Therapeutics Co., Ltd, China) from Day 10 to Day 17. Subsequent photoreceptor commitment was performed using NouvNeu Neural Differentiation Medium supplemented with 0.5 μM AM580 (Selleck Chemicals LLC, USA) from Day 17 to Day 24. Differentiated PPCs were cryopreserved in STEM-CELLBANKER medium (Youkang Biotechnology Co., Ltd, China).

#### Flow cytometry

Cells in a 6-well plate were treated with Accutase digestion solution and incubated at 37°C for 5–8 min. Digestion was terminated by adding an equal volume of hNeuron medium, followed by gentle pipetting to generate a single-cell suspension. After centrifugation, the supernatant was discarded, and the cells were resuspended in ice-cold PBS. The cell concentration was adjusted to 1 × 10^6^ cells/mL using a hemocytometer or automated cell counter. Following another centrifugation step, cells were fixed with 4% PFA at 4°C for 15 min, permeabilized with 0.1% Triton X-100 (TRI) at room temperature (RT) for 10 min, and blocked with 4% BSA in PBS at RT for 30 min. Primary antibodies diluted in blocking buffer were incubated with the cells at 4°C for 1 h. After three washes with PBS, fluorophore-conjugated secondary antibodies were applied and incubated at RT for 30 min in the dark. Cells were washed twice with PBS, filtered through a 200-μm nylon mesh to remove aggregates, and transferred to flow cytometry tubes for analysis using a calibrated flow cytometer.

#### scRNA-seq

The cell sample was collected at Day 24 of differentiation for single-cell RNA sequencing. Raw PPCs sequencing data were first processed using 10x Genomics’ Cell Ranger pipeline (version 7.1) for demultiplexing, alignment to the GRCh38 human genome, and generation of gene-cell count matrices. The resulting count matrices were imported into R and analyzed with the Seurat package (v5.0.1). Quality control filtering excluded cells with fewer than 200 detected genes (nFeature_RNA <200) or more than 5% mitochondrial gene content (percent.mt > 5%), Individual datasets were merged using Seurat’s merge function for integrated downstream analyses including normalization, scaling, and dimensionality reduction. For comparative analysis, PPCs data were integrated with the established human fetal retinal development scRNA-seq reference atlas.^24^ Batch effect mitigation was achieved through implementation of the FastMNN algorithm. Preprocessing involved normalization using the LogNormalize method with scaling to 10,000 unique molecular identifier (UMI) counts per cell. Integration of datasets was conducted using Seurat IntegrateLayers function incorporating the FastMNNIntegration computational framework. Dimensionality reduction was performed using uniform manifold approximation and projection (UMAP) based on the top 30 principal components, followed by neighborhood graph construction and clustering using standard Seurat workflows.

#### Bulk RNA-seq

To further characterize the developmental trajectory and lineage commitment of PPCs beyond the limitations of single-cell transcriptomics at Day 24, bulk RNA-seq was performed on PPC-derived populations at Day 54. Cells were harvested and total RNA extracted using the RNeasy Mini Kit (Qiagen), with RNA integrity verified (RIN >8.0) via Agilent Bioanalyzer. Stranded mRNA libraries were prepared from 500 ng total RNA per sample using the NEBNext Ultra II Directional RNA Library Prep Kit and sequenced on an Illumina NovaSeq 6000 platform to generate about 50 million 150-bp paired-end reads per sample. Raw reads underwent quality control with FastQC, followed by quality filtering and adapter trimming with fastp. Clean reads were then aligned to the GRCh38 human genome using hisat2. Gene-level quantification was performed against Ensembl release 109 annotations using featureCounts. Critical lineage markers were interrogated: photoreceptor commitment was assessed through expression of GNB1, PDE6B, and GNB3; and RGC identity was evaluated via POU4F2 and SHH expression. Transcriptional profiles were benchmarked against established human fetal retinal development trajectories to validate alignment with late retinogenesis.

#### Immunofluorescence staining of cells

Sterilized glass coverslips were placed in a 24-well plate, and cells were seeded at a density of 1×10^5^ cells/mL. After 24 h of culture, the medium was aspirated, and cells were gently washed twice with ice-cold PBS. Cells were fixed with 4% PFA at RT for 15 min, followed by permeabilization and blocking in a solution containing 4% BSA and 0.5% TRI in PBS at RT for 1 h. Primary antibodies diluted in blocking buffer were applied and incubated overnight at 4°C. After three washes with PBS, fluorophore-conjugated secondary antibodies were added and incubated at RT for 2 h in the dark. Nuclei were counterstained with 1 μg/mL DAPI for 5 min at RT, protected from light. Following final PBS washes, coverslips were mounted with antifade mounting medium and stored in the dark. Images were acquired using a laser scanning confocal microscope (Zeiss LSM 880) equipped with ZEN imaging software.

#### Preparation of cells for transplantation

All transplanted PPCs originated from a single differentiation batch and were pre-calibrated before freezing to a concentration of 1×10^6^ cells/mL in standardized cryopreservation medium, aliquoted into individual cryovials (1 mL/vial). The cryopreserved cells were retrieved from the liquid nitrogen tank and rapidly thawed in a 37°C water bath. The thawed cells were transferred into DMEM medium (Grand Island Biological Company, USA) and incubated at RT for 5 min. Cell count and post-thaw viability were determined using trypan blue staining solution, consistently exceeding 98%. After centrifugation to remove the supernatant, the cell pellet was resuspended in DPBS (Grand Island Biological Company, USA) according to the counting results, adjusting the cell concentration to 2×10^5^ cells/μL. The resuspended cell suspension was maintained on ice and used within 5 h.

#### SRI

Mice were anesthetized via inhalation of isoflurane (RWD Life Science Co., Ltd., China). The periocular area was disinfected with iodophor, followed by instillation of compound tropicamide eye drops onto the cornea for pupillary dilation, which was allowed to proceed for 3 min. Subsequently, proparacaine hydrochloride eye drops were applied to the cornea for topical ocular anesthesia, with a 1-min equilibration period. The mouse was then immobilized on the surgical platform, positioning the target eye upward. A lens was placed on the ocular surface, and 1% sodium carboxymethyl cellulose eye drops were applied. The mouse’s head position was adjusted until the retinal vasculature was clearly visualized. A 30-gauge insulin needle was inserted at the corneal limbus to create a corneal incision. A 33-gauge needle attached to a microinjector (Hamilton Company, Switzerland) was loaded with 1 μL of cell suspension or DPBS, advanced through the incision until the tip reached the supraretinal space, and used to puncture a small opening in the retina. Cell suspensions were infused into the SRS, which is an immune-privileged niche between the retinal pigment epithelium and photoreceptor layer known to support graft survival. The cell suspension was slowly infused into the SRS. The needle was retained in place for 1 min post-injection to minimize extensive retinal detachment and then withdrawn gently along the insertion trajectory. After a 3-min observation period under a surgical microscope, fundus examination was performed to confirm procedural success. Postoperative topical application of levofloxacin ophthalmic ointment was administered to the eye. Crucially, to minimize technical variability, each transplantation session involved mice from all three experimental groups (C57, MNU, and rd10) receiving cells from the same cryovial. All procedures were performed by the same operator using identical surgical instruments within the strict 5-h post-thaw window.

#### IVI

Mice were anesthetized via inhalation of isoflurane. The periocular area was disinfected with iodophor, followed by instillation of compound tropicamide eye drops onto the cornea for pupillary dilation, which was allowed to proceed for 3 min. Subsequently, proparacaine hydrochloride eye drops were applied to the cornea for topical ocular anesthesia, with a 1-min equilibration period. The mouse was then immobilized on the surgical platform, positioning the target eye upward. The 33 needle attached to the microsyringe uploaded 1 μL cell suspension into the vitreous cavity (VC) after making an incision in the corneosclera margin. The cell suspension was slowly infused into the VC. The needle was retained in place for 1 min post-injection and then withdrawn gently along the insertion trajectory. Postoperative topical application of levofloxacin ophthalmic ointment was administered to the eye.

#### OCT

Mice were anesthetized with isoflurane inhalation until reaching a surgical plane of anesthesia, characterized by steady respiratory rhythm and muscle relaxation. The mouse was transferred to a stereotaxic surgical platform, and anesthesia was maintained via a nose cone connected to the isoflurane delivery system. Compound tropicamide eye drops were instilled onto the cornea for pupillary dilation and allowed to act for 3 min. To prevent corneal dehydration, 0.5% sodium carboxymethyl cellulose eye drops were applied to moisten the ocular surface. The anesthetized mouse was then secured on a stereotaxic animal platform, with the eye positioned orthogonally to the OCT probe. The Robotrack OCT software was launched, and the “Mouse Retina” preset protocol was selected. Scanning parameters were set as follows: depth range 1.5–2.5 mm, high-resolution mode, and high-speed acquisition mode to minimize motion artifacts. The OCT probe was aligned to the central axis of the eye using a three-axis motorized stage. Real-time preview imaging was activated, and the focal plane was adjusted until distinct retinal layers were visualized. The fundus reflex was utilized to guide alignment, ensuring the optical axis coincided with the ocular center. Either “B-scan” or “Volume scan” mode was selected, with the scanning area covering the optic nerve head or region of interest. Images were acquired and saved for subsequent analysis.

#### Immunofluorescence staining of cryosectioned retinal tissue

Mice were humanely euthanized by cervical dislocation in compliance with institutional animal ethics guidelines. The eyes were rapidly enucleated and immersed in ice-cold 4% PFA for 1 h at 4°C. The ocular globes were circumferentially incised along the corneal limbus using ophthalmic scissors, and the lens and vitreous body were carefully removed. Fixed retinal tissues were rinsed in PBS and sequentially dehydrated in a graded sucrose series (10%, 20%, and 30% w/v in PBS) at 4°C until equilibrated. The dehydrated retinas were embedded in OCT compound, flash-frozen in liquid nitrogen, and stored at −80°C for long-term preservation. Serial 14 μm-thick retinal cryosections were cut along the optic nerve axis using a cryostat and air-dried at RT for 30 min. Sections were sealed in moisture-proof containers and stored at −20°C until staining. Prior to staining, slides were equilibrated to RT for 10 min to minimize thermal stress. Sections were permeabilized and blocked with 0.5% TRI and 4% BSA in PBS at RT for 1 h. Primary antibodies diluted in blocking buffer were applied and incubated overnight at 4°C. After three PBS washes, fluorophore-conjugated secondary antibodies were incubated at RT for 2 h in the dark. Nuclei were counterstained with 1 μg/mL DAPI for 5 min at RT, protected from light. Following final PBS washes, sections were mounted with antifade mounting medium and coverslipped. Images were acquired using a laser scanning confocal microscope (Zeiss LSM 880) and a high-content fluorescence imaging system (Leica Thunder Imager) with ZEN or LAS X software.

#### TEM

Three months post cell transplantation, mice were euthanized and the eyes were enucleated. The harvested tissues were fixed in 2.5% glutaraldehyde (Absin Bioscience Inc., China), post-fixed in osmium tetroxide (SPI Supplies Structure Probe, Inc., USA), dehydrated through a graded series of ethanol (Sinopharm Chemical Reagent Co., Ltd., China) and acetone (Sinopharm Chemical Reagent Co., Ltd., China), and embedded in epoxy resin (SPI Supplies Structure Probe, Inc., USA). Ultrathin sections (70–90 nm) were prepared, stained with aqueous uranyl acetate (SPI Supplies Structure Probe, Inc., USA) and lead citrate (Sinopharm Chemical Reagent Co., Ltd., China), and subsequently examined using a TEM.

#### FVEP

Mice were anesthetized, and electrodes were implanted epidurally over V1. After overnight dark adaptation, FVEPs were recorded in a darkroom using white flash stimuli (5.0 cd/m^2^, 2800 μs, 64 trials). Signals were sampled at 2000 Hz, and N1/P1 amplitudes were analyzed using RetiMINER 4.0.

#### OMR

Visual acuity was assessed using a four-monitor system displaying rotating gratings (0.10–0.60 cycles/degree). Head movements were tracked, and the highest spatial frequency eliciting a response was recorded as visual acuity.

#### Light/dark box test

Dark-adapted mice were placed in a two-compartment box (18 × 20 × 18 cm) and allowed to explore for 5 min. The time spent in the dark compartment was recorded and analyzed.

### Quantification and statistical analysis

#### Statistical analysis

All statistical analyses were performed using Prism software (GraphPad Prism version 8.0.2). Data are presented as mean ± standard error of the mean (SEM). For each experiment, the symbol “n” in figures denotes the sample size, which corresponds to independent biological replicates unless explicitly stated. All reported values are derived from at least three independent experiments. Statistical significance between groups was determined by one-way analysis of variance (ANOVA) followed by appropriate post hoc tests. The two-tailed unpaired *t* test was used to determine statistical significance between two groups. A probability threshold of *p* < 0.05 was considered statistically significant. Significance levels are denoted as follows: not significant (n.s.), *p* < 0.05 (∗), *p* < 0.01 (∗∗), *p* < 0.001 (∗∗∗), and *p* < 0.0001 (∗∗∗∗).
