# Supplementary material for: Efficient derivation of hiPSC-derived photoreceptor precursor cells and their neuroprotective effects in retinal degeneration
Source: iScience. 2025 Nov 24;28(12):114196. doi: 10.1016/j.isci.2025.114196 (PMC12744287; doi:10.1016/j.isci.2025.114196)
Supplement: Document S1. Figures S1–S3 [file mmc1.pdf]

**iScience, Volume *28***

## Supplemental information

# Efficient derivation of hiPSC-derived photoreceptor precursor cells and their neuroprotective effects in retinal degeneration

**Yuxin Du, Jingjing Cao, Lumeng Niu, Gao Tan, Jingmin Zhang, Xiaoqian Yi, Yu Li, Jun Wei, and Yin Shen**

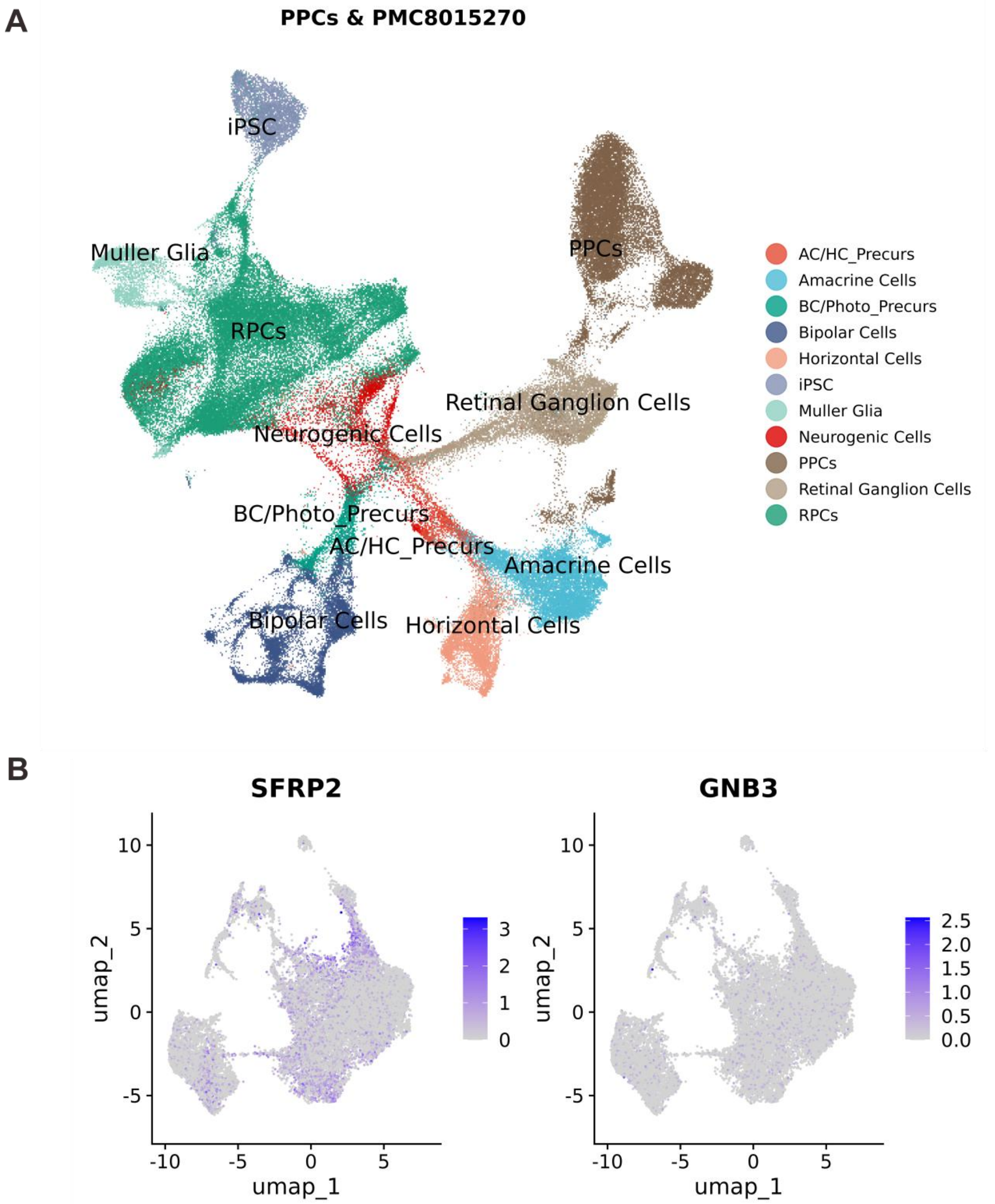


Supplemental Figure 1. Characterization of hiPSC-derived PPCs at Day 24 of differentiation via scRNA-seq.

(A) UMAP of integrated scRNA-seq data from Day 24 PPCs and a human fetal retinal reference atlas. The PPCs cluster localizes near RGCs in the embedding but represents a distinct transitional precursor population. (B) Expression of SFRP2 (early RPC marker) across PPCs. Widespread expression confirms an immature developmental state. (C) Absence of GNB3 (late-stage photoreceptor marker) in PPCs,

supporting their earlier developmental status relative to late-fetal reference cells.

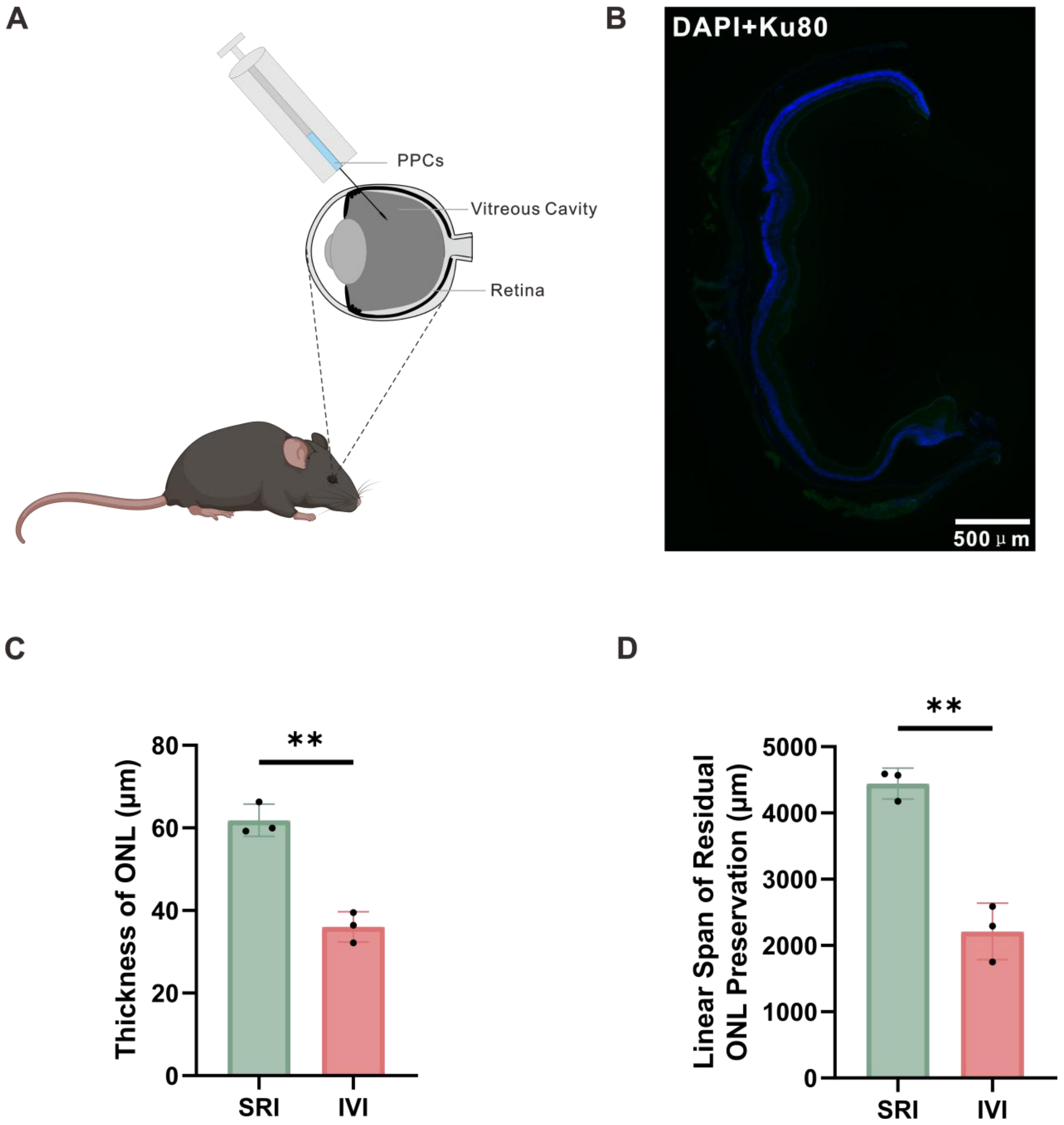


Supplemental Figure 2. Comparative analysis of different administration routes on the neuroprotective efficacy of transplanted PPCs in MNU-induced mice.

(A) Schematic of IVI. (B) Absence of $Ku80^+$ human donor cells in the VC and partial ONL preservation in MNU-modeled mice 1 month after IVI transplantation. Scale bar represents 500 μm. (C) Comparative analysis of average ONL thickness between SRI and IVI groups ($n \geq 3$). (D) Spatial distribution analysis of ONL protective coverage in SRI and IVI groups. Data are represented as mean ± SEM (two-tailed unpaired t test; $^{**}p < 0.01$).

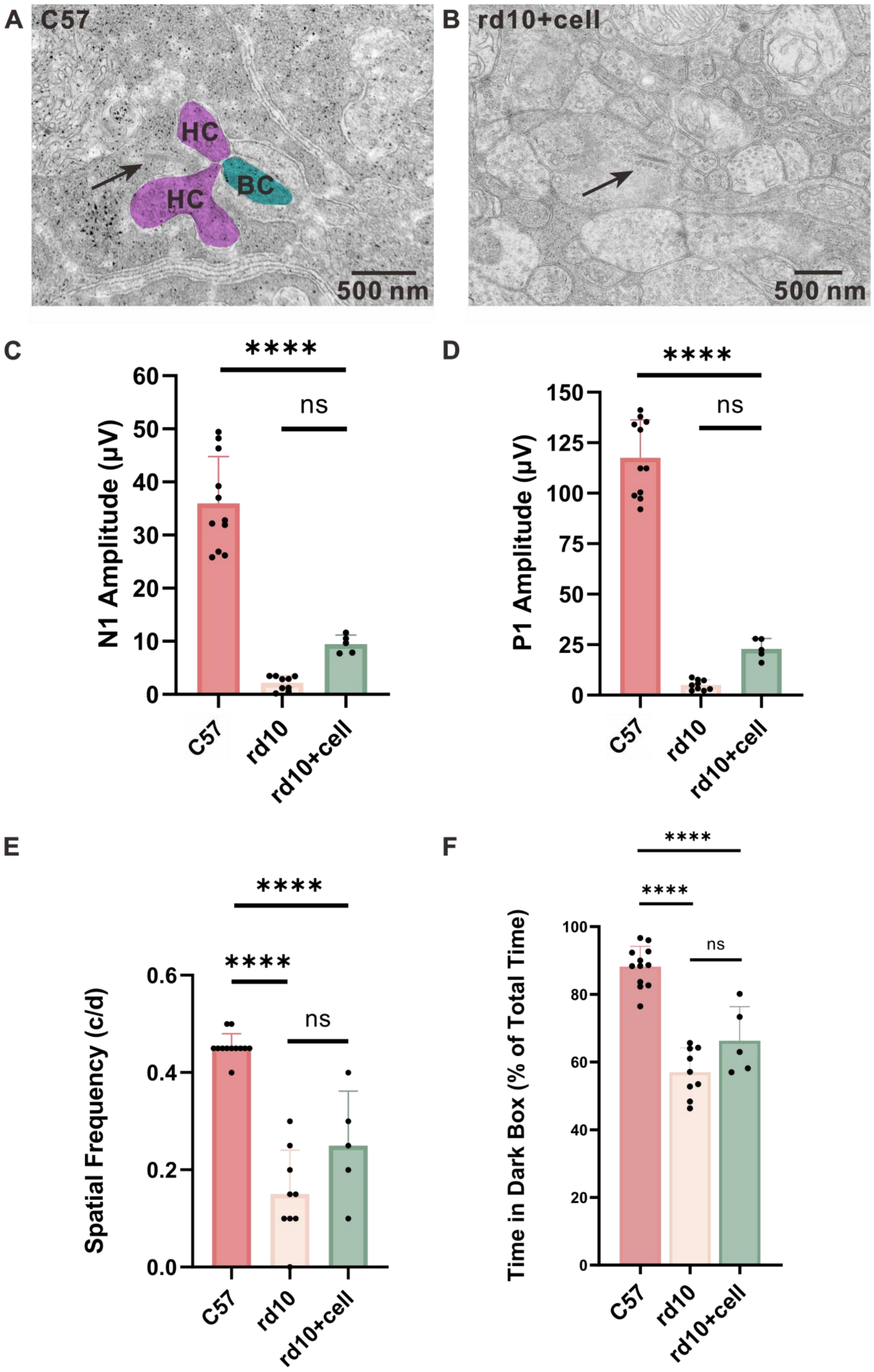


Supplemental Figure 3. Analysis of Synaptic Ultrastructure and Visual Function in rd10 Mice Post-Transplantation.

(A) TEM revealed characteristic synaptic triads in the retinas of C57 mice, composed of ribbon synapses (arrow), BCs, and horizontal cells (HCs). Scale bar represents 500 nm. (B) Although typical synaptic triads were not observed in the retinas of rd10 + cell mice, ribbon synapses (arrow) were still detectable. Scale bar represents 500 nm. (C-D) FVEPs were recorded in rd10 + cell mice (n = 5) and rd10 controls (n = 9). Although the rd10 + cell group showed an increasing trend in N1 and P1 wave amplitudes, the differences were not statistically significant. (E) In the OMR assessment, the rd10 + cell group (n = 5) demonstrated an increasing trend in the mean visual acuity compared to the rd10 group (n = 9); however, this difference did not reach statistical significance. (F) In the light/dark box test, the rd10 + cell group (n = 5) exhibited a trend toward an increase in the mean time spent in the dark chamber compared to the rd10 group (n = 9); however, this difference lacked statistical significance. Data are represented as mean ± SEM (one-way ANOVA with appropriate post hoc tests; n.s., not significant, ****$p < 0.0001$).
